# The irrepressible influence of vocal stereotypes on trust

**DOI:** 10.1177/17470218231211549

**Published:** 2023-11-28

**Authors:** Ilaria Torre, Laurence White, Jeremy Goslin, Sarah Knight

**Affiliations:** 1 Division of Interaction Design and Software Engineering, Chalmers University of Technology, Gothenburg, Sweden; 2 KTH Royal Institute of Technology, Stockholm, Sweden; 3 Newcastle University, Newcastle upon Tyne, UK; 4 University of Plymouth, Plymouth, UK; 5 University of York, York, UK

**Keywords:** Accent, prosody, trust

## Abstract

There is a reciprocal relationship between trust and vocal communication in human interactions. On one hand, a predisposition towards trust is necessary for communication to be meaningful and effective. On the other hand, we use vocal cues to signal our own trustworthiness and to infer it from the speech of others. Research on trustworthiness attributions to vocal characteristics is scarce and contradictory, however, being typically based on explicit judgements which may not predict actual trust-oriented behaviour. We use a game theory paradigm to examine the influence of speaker accent and prosody on trusting behaviour towards a simulated game partner, who responds either trustworthily or untrustworthily in an investment game. We found that speaking in a non-regional standard accent increases trust, as does relatively slow articulation rate. The effect of accent persists over time, despite the accumulation of clear evidence regarding the speaker’s level of trustworthiness in a negotiated interaction. Accents perceived as positive for trust can maintain this benefit even in the face of behavioural evidence of untrustworthiness.

## Introduction

Trust is fundamental to human society (e.g., Baier, 1986). Because we establish social relationships primarily through linguistic interaction, trust and language are mutually dependent: our spoken assertions can serve as signals of trustworthiness, but—at the same time—verbal interactions work because underlying reciprocal trust is assumed (e. g. the cooperative principle, Grice, 1975). Indeed, as with other low-cost signals in animal communication, the symbolic system of language maintains its social utility through the availability of external demonstrations of reliability (Lachmann et al., 2001), even if such demonstrations are only occasionally required in practice.

The higher the degree of mutual self-interest between parties in a verbal interaction, the lower the demand for such external validation: Thus, we tend to implicitly trust the speech of our family, unless evidence suggests otherwise (e.g., the “mother tongues” hypothesis, Fitch, 2004). By contrast, strangers may be suspected as “free-riders” (e.g., Doebeli et al., 2004). Because speech is such a powerful indicator of social origins, this entails a strong bias towards favouring speakers with familiar accents (e.g., Bestelmeyer et al., 2014).

Accent is not the only verbal cue to trustworthiness, however: Prosody and idiolectal acoustic-phonetic features are also exploited in such judgements (e.g., Apple et al., 1979). Furthermore, people make fast and consistent trustworthiness judgements upon hearing someone’s voice for the first time (McAleer et al., 2014). What is less clear is how initial impressions based on speech cues change through extended interaction and how they are affected by evidence of trustworthiness provided by the speaker’s behaviour. One hypothesis is that the power of voice-based cues to trustworthiness diminishes as direct experiential evidence becomes available. This would be in line with findings from the face perception literature: For example, Chang et al. (2010) reported a gradual decrease in the weighting of facial trait information compared with behavioural evidence of trustworthiness over the course of a virtual interaction. Here, we examine the dynamics of trust judgements: Using an iterated investment game, we test whether initial implicit appraisal based on vocal characteristics—in particular, accent and prosody—survives behavioural demonstration of a speaker’s trustworthiness.

Accents have often been associated with personality judgements, including trustworthiness. Although listeners are not particularly effective at explicit accent identification (Goggin et al., 1991), dialectal and idiolectal perceptions still shape their implicit judgements of the speaker. For example, native accents tend to be perceived as more trustworthy than non-native accents (Lev-Ari & Keysar, 2010). Furthermore, within the British Isles, Standard Southern British English (SSBE) tends to be rated as more pleasant and prestigious than regional accents (Bishop et al., 2005), and countryside accents like Devon are often rated as more friendly and trustworthy than city accents like London or Birmingham (Bishop et al., 2005). Apart from SSBE, British accents index relatively restricted geographical regions, and evaluations of accent labels are likely to be influenced by stereotypes based on regional socio-economic perceptions (Giles, 1970).

Specific acoustic-phonetic features, such as prosody, also influence personality judgements. Studies are, however, strikingly inconsistent about how prosodic dimensions influence trust. On one hand, male and female embodied conversational agents were trusted less when the agents spoke with higher pitch (Elkins & Derrick, 2013). Similarly, Apple et al. (1979) found that male speakers with a high pitch and slow speech rate were rated as “less truthful.” Moreover, men with intrinsically lower pitch and higher pitch variation were found to actually cooperate more in a public goods game (Tognetti et al., 2020). In line with that, speakers of both genders have also been shown to raise their pitch when lying (Villar et al., 2013). In apparent contrast, however, male and female actors communicated sarcasm through lower pitch and slower speech rate (Cheang & Pell, 2008); sincerity, for male and female voices, has been encoded in synthetic speech using greater pitch range and faster articulation rate (Trouvain et al., 2006). Slow speech rate in male speakers has been associated with incompetence (Smith et al., 1975), but also with charisma and persuasion (Niebuhr et al., 2016). Finally, voice quality—such as breathiness or creakiness—can also convey personality information: For example, Blood et al. (1979) found that women with hypernasal or breathy voices were rated as less attractive on various personality dimensions.

Some of these inconsistencies may derive from methodology. Most studies assess personality attributions through questionnaires, which typically require participants to rate items on Likert-type scales along various dimensions. However, such explicit attitudinal data do not correlate with behaviour (Greenwald, 1990); this issue, sometimes called the “attitude-behaviour problem,” was originally identified in Wicker’s (1969) literature review. Several studies reported therein found a discrepancy between racial prejudice (in 1960’s America) and actual behaviour towards people belonging to different ethnicities. Thus, listeners’ explicit voice-based trust ratings may not strongly predict whether they will trust particular speakers in natural encounters. Furthermore, questionnaires only focus on immediate impressions and cannot determine whether these voice-based attributions survive experience with speakers’ actual behaviour.

Consequently, we chose a game-theoretic approach that allows the speaker–listener relationship to be tracked dynamically, as the listener’s original attributions are confronted with the speaker’s actual behaviour. An advantage of this approach is that the behavioural indices of trustworthiness can be manipulated in an experimental context, such as the “investment game.” In this game, the participant receives a sum of money and can invest some or all of it with another partner, trusting that they will return more than the original investment. The sum of money invested by the participant provides an implicit measure of trust in the partner (Berg et al., 1995). Simulated investments in this game have been used as a proxy for trust as influenced by various attributes, including gender (Chaudhuri & Gangadharan, 2007) and facial expression (Krumhuber et al., 2007). The method has rarely been used to examine trustworthiness attributions to voices, although Knight et al. (2021) failed to find an impact of “cheerful” versus “neutral” voices on investment patterns.

Participants played an iterated investment game with a simulated partner, via audio only (the virtual partner was not visually represented). In this paradigm, the trustworthiness of the game partner is demonstrated in how much money they return to the participant, with trustworthy partners returning more money than the participant’s original investment and untrustworthy partners returning less. The voices of our simulated partners were recorded by speakers of four British accents, with reference to the accent attitudes literature (Bishop et al., 2005; Giles, 1970): SSBE and Plymouth, previously associated with relatively high trust, and London and Birmingham, with lower trust. Furthermore, we recorded three different speakers of each of these accents, to also examine the effect of idiosyncratic prosodic differences. By playing multiple rounds of the game, we aim to establish how first impressions are modified as participants experience their partners’ behaviour. It might be expected that the influence of accents on trustworthiness attributions, as indexed by money returned to the partner (the dependent variable in the investment game), would diminish with experience of actual behaviour. However, our findings—as presented below—suggest that the accent influences on trust persist throughout the task.

## Method

### Participants

Participants were 84 native British English speakers (52 females, 32 males) aged 18-67 (median = 21, *SD* = 11). They were undergraduate university students who received course credit for participation, or members of the public who received a small honorarium. They all provided written consent for their data to be collected, in accordance with the University of Plymouth ethics guidelines. Their geographic origins were reported as southwest England (*N* = 44), southeast England (*N* = 20), Midlands (*N* = 7), Wales (*N* = 5), northwest England (*N* = 3), East Anglia (*N* = 2), and northeast England (*N* = 1). Only participants with a U.K. English language background were included in the study, resulting in one other participant being eliminated. The questionnaire data for one participant were not recorded due to a technical error, so that we had data from 83 participants in the investment game, and from 82 participants in the postexperiment questionnaire.

### Stimuli

We recorded 12 female native English speakers (all in their early 20s) for the voices of the virtual game partner, three speaking with each of the following accents: Plymouth, Birmingham, London, and SSBE. Other female speakers were recorded for a postexperiment questionnaire. One was a native English speaker with a Belfast accent and five were second language (L2) English speakers, whose native languages were Austrian German (Linz), French (Paris), Italian (Naples), Greek (Cyprus), and Mandarin (Taipei). Spoken samples from each speaker were assessed by phonetics experts to ensure that accent features were present.

Because each participant played four 20-round games with four different virtual game partners, each speaker was required to read four different blocks of 20 sentences (listed in Appendix A). Sentences were approximately matched in terms of syllable number (*M* = 16.95 syllables, *SD* = 1.08). The recorded utterances were amplitude-normalised and a noise-removal filter was applied.

Prosodic characteristics of each utterance were measured to characterise idiosyncratic differences between speakers and accents (Table 1). Segmentation and labelling of utterances were done with the MAUS General Web service forced alignment tool Schiel (2015). The segmentation thus obtained was used to extract prosodic measures—mean pitch, pitch range, voice quality, and articulation rate—in Praat Boersma and Weenink (2017) and MATLAB. Mean pitch was calculated as mean f0 value for each vowel and then averaged for individual utterances. Pitch range was calculated as the difference between the 10th and 90th percentiles of f0 value for each vowel (to eliminate potential outliers, Patterson & Ladd, 1999), and then averaged for individual utterances. Articulation rate was calculated as syllables/second, excluding pauses (Jacewicz et al., 2009). We used H1 – H2—the difference between the first and second harmonic for each vowel—as a measure of voice quality (Johnson, 2002), using VoiceSauce (Shue et al., 2011).

**Table 1.** Means for prosodic measures by accent (*SD* in parentheses).

	SSBE	Plymouth	London	Birmingham
f0 (Hz)	221.3 (11.8)	217.7 (21.1)	202.8 (17.8)	204.7 (32.6)
f0 range (Hz)	33.7 (10.6)	28.1 (8.3)	31.6 (11.0)	28.3 (9.8)
Articulation rate (syll/s)	4.2 (0.5)	3.9 (0.5)	4.0 (0.5)	3.8 (0.7)
H1 – H2 (dB)	9.2 (1.6)	8.5 (1.4)	8.7 (1.3)	7.1 (1.9)

### Procedure

Participants were told that the goal of the game was to earn as much money as possible, and that mutual co-operation with the game partner would lead to greater profit. They were informed that they could not verbally interact with the partner, but they would hear the game partner speak an utterance at the beginning of each round. The participant started each of the 20 rounds with a notional sum of £8, and she or he then had to decide to invest all, part, or none of the £8 with the game partner. Whatever was invested, the game partner received 3 times the invested amount. The game partner was programmed with one of two behaviours regarding how much they returned to the participant: *generous*—returning between 120% and 240% of the investment; *mean*—returning between 0% and 120%. For both conditions, the pattern of return percentages was randomly determined in advance for each of the 20 rounds, and the same *generous* or *mean* patterns were always used. For example, the *generous* partner always returned 150% of the investment in Round 1, 150% in Round 2, 180% in Round 3, 120% in Round 4, and so on.

Each participant played two games with a *generous* virtual partner and two with a *mean* virtual partner, with the order of behaviours counterbalanced across participants. Each participant heard all four accents, with speaker–accent pairing systematically varied between participants. A different block of 20 utterances was used for each game, with block–accent pairings also varied between participants within conditions. Thus, there was a 4 (accent: SSBE, Plymouth, Birmingham, London) by 2 (behaviour: *generous* or *mean*) within-subject design, with the pairing of accents and behaviours counterbalanced between participants.

Each round of the game proceeded as follows: (1) Participants heard the utterance from the virtual partner; (2) participants indicated by pressing a digit key how much of £8 they wished to invest, in integers from 0 to 8; and (3) participants saw a summary screen of the monetary transactions to and from the virtual partner during the round. This summary included the amount that the virtual partner had returned to them and also showed the total money that they had accumulated over all of the rounds so far.

After finishing all four 20-round games, participants completed a short on-screen questionnaire. First, they were asked to identify the accents that they heard in the game. Then, participants heard two utterances from each of the 10 recorded accents (including the L2 accents) in random order, and rated the voices on a 7-point Likert-type scale (1 = *very untrustworthy*, 7 = *very trustworthy*). In this task, for the four virtual partner accents, participants heard different speakers than the ones they heard in the game. Finally, participants completed a questionnaire about their age, gender, region of origin, and what accent they spoke. The experiment lasted approximately 25 minutes.

## Results

To determine the effects of game behaviour and vocal characteristics on investments, linear mixed-effects models were fitted to the data in two successive stages. The first stage was a confirmatory analysis testing the hypothesised effects of interest (behaviour—*generous/mean*; accent; game turn). This confirmatory analysis used a backward stepwise procedure, in which nonsignificant effects were systematically removed to keep the model as parsimonious as possible. The initial model contained all possible fixed effects and their interactions. These fixed effects were then tested for their contribution to model fit using likelihood ratio tests, progressing from the most complex level (i.e., the three-way interaction) to the least complex. The order of effect removal within a given level was determined by Akaike information criterion (AIC) values: In each case, the reduced model producing the greatest drop in AIC was used as the new baseline for further nested comparisons. The final model was then used as the baseline for the second stage, which was an exploratory analysis testing contributions from the measured prosodic features (mean f0; f0 range; articulation rate; H1 – H2). This exploratory analysis used a forward stepwise procedure, selecting each successive predictor according to the lowest AIC value. In all models, investment was the dependent variable and random intercepts were included for participant and sentence set. All analyses were performed in R version 4.1.1; models were fitted using the *lme4* package and post hoc tests were carried out using the *emmeans* package; all post hoc comparisons were Tukey-corrected and used the Kenward–Roger approximation for degrees of freedom.

### Confirmatory analysis

The final model included main effects of behaviour, game turn and accent, and significant interactions of Behaviour × Accent and Behaviour × Game Turn. The main effect of behaviour, $\chi^{2}(1)=345.61,p<.001$ , indicated higher investments to *generous* virtual partners, with an average investment of £6.14 to *generous* virtual partners and £2.40 to *mean* virtual partners. The main effect of game turn, $\chi^{2}(1)=9.45,p<,001$ , indicated higher overall investments as the game progressed. The main effect of accent, $\chi^{2}(3)=13.05,p<.01$ , indicated that investments were highest for SSBE speakers. Post hoc pairwise comparisons showed that investments to SSBE speakers were higher than to any other accent type (all p<.001 ). No other pairwise comparisons reached significance.

**Figure 1.**
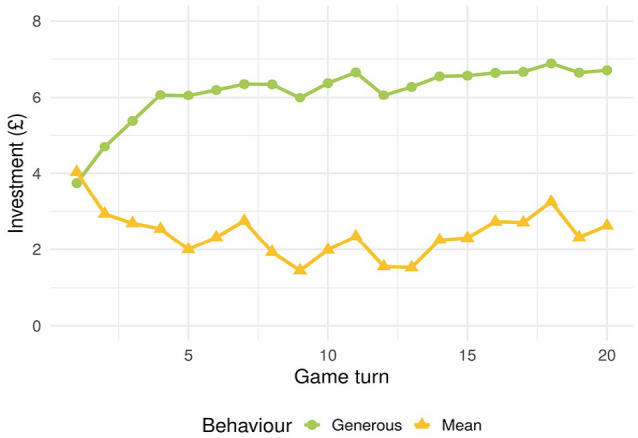
Average investments in the *generous* and *mean* condition according to game turn.

The interaction between behaviour and game turn, $\chi^{2}(1)=201.03,p<.001$ , indicated that investments increased in the *generous* condition and decreased in the *mean* condition as the game progressed. These effects of behaviour and game turn are summarised in Figure 1. The interaction between behaviour and accent, $\chi^{2}(3)=12.74,p=.005$ , indicated that that the investments in the *generous* condition were significantly higher for SSBE speakers than for speakers from Plymouth, London, and Birmingham (all p<.01 ). By contrast, in the *mean* condition, investments to SSBE and London speakers were higher than the investments to Birmingham speakers (both p<.05 ), but no other pairwise comparisons reached significance. However, examination of the descriptive statistics (Table 2) indicated that some of these pairwise comparisons may have been misleading: The estimated marginal means generated by the *emmeans* package diverged slightly in some cases from the observed means, thus leading to implausible significant differences. Specifically, the significant difference generated by *emmeans* between the SSBE and Birmingham speaker in the *generous* condition seemed unlikely given the nearly identical observed means. To exclude the possibility of a truly significant difference in this case, we ran an independent-samples *t* test to compare investments between the SSBE and Birmingham speakers in this condition. It should be noted that the data were not entirely independent since, due to counterbalancing, a small number of participants heard both the SSBE and Birmingham speakers in the *generous* condition. However, the majority of the datapoints were from unique participants, hence our choice of an independent-samples test. This *t* test indicated a nonsignificant difference as expected, t(80)=−0.02,p=.98 . Thus, the interaction in the main model between behaviour and accent should be interpreted as indicating that the investments in the *generous* condition were significantly higher for SSBE speakers than for speakers from Plymouth and London, but not significantly different to speakers from Birmingham.

**Table 2.** Means and *SD* of investments to the four target accents (*SD* in parentheses).

	Overall	*Generous*	*Mean*
SSBE	4.55 (2.86)	6.29 (2.11)	2.72 (2.37)
Plymouth	4.17 (2.90)	6.01 (2.05)	2.41 (2.48)
London	4.26 (2.96)	5.98 (2.28)	2.45 (2.47)
Birmingham	4.14 (2.96)	6.29 (2.06)	2.09 (2.10)
*Birmingham minus Speaker 1*	*4.17 (3.03)*	*6.53 (1.79)*	*1.99 (2.19)*

The bottom italicised row shows means and *SD* of investments in the Birmingham accent with Birmingham Speaker 1 removed from the data set.

**Figure 2.**
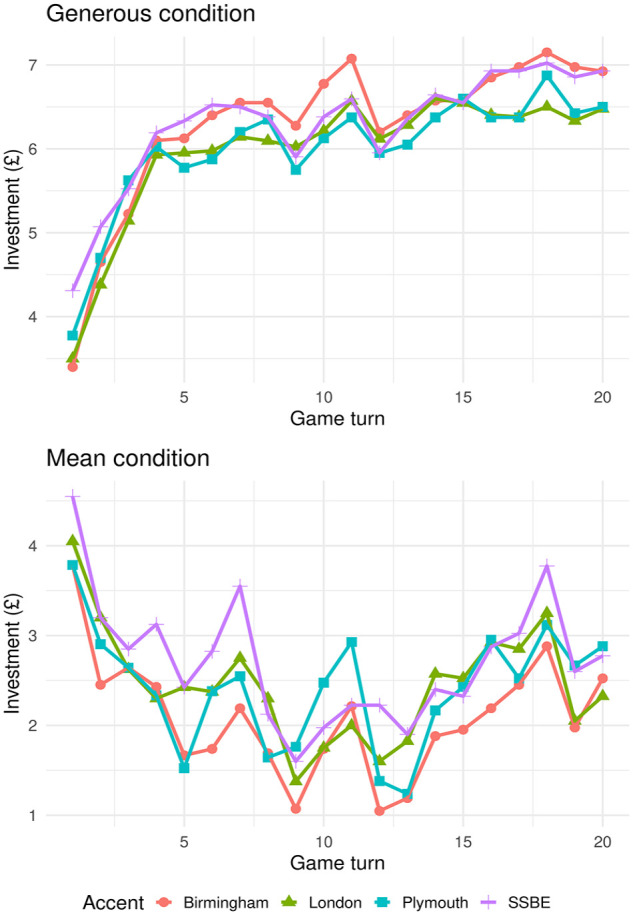
Average investments to the four target accents in the *generous* (top figure) and *mean* (bottom figure) conditions.

There was no interaction between accent and game turn, $\chi^{2}(3)=2.85,p=.42$ . Despite participants’ increasing experience of the actual behaviour of game partners, the effect of accent on judgements of trustworthiness persisted. As the interaction between accent and behaviour shows, the persistent accent effect is mediated by a re-ranking of trustworthiness of accents according to overall behaviour (Table 2). However, even after 20 rounds in which overall behaviour has hugely impacted participant investment (Figure 1), the partner’s accent continues to affect levels of investment (Figure 2).

### Exploratory analysis

The forward stepwise procedure resulted in a final exploratory model which included a main effect of mean f0 and an interaction of Mean f0 × Game Turn, in addition to the effects present in the final confirmatory model (behaviour; game turn; accent; Behaviour × Accent; Behaviour × Game Turn). The main effect of mean f0 indicated that a higher mean vocal f0 of the virtual partner was associated with higher investments, $\chi^{2}(1)=5.94,p<.05$ . The interaction between mean f0 and game turn, $\chi^{2}(1)=13.61,p<.001$ , is harder to interpret, but as indicated below, removing one prosodically-outlying speaker changed the results of the exploratory analyses, so we refrain from further discussion here.

### Reanalysis without Birmingham Speaker 1

**Figure 3.**
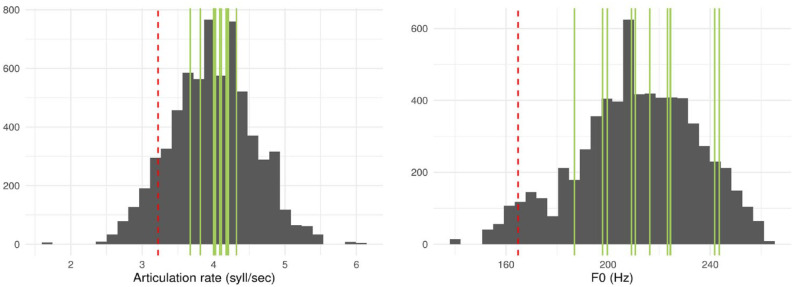
Distribution of articulation rates (left) and f0s (right) across all the utterances of the speakers used in the game. The red, dashed vertical lines are the mean value of speaker Birmingham 1, while the green vertical lines are the mean values of all the other speakers.

Birmingham Speaker 1 had by far the slowest articulation rate (Figure 3, left plot), as well as the lowest average f0 (Figure 3, right plot). In the distributions of mean articulation rate and f0 by speaker, Birmingham 1 is the only speaker to fall more than one standard deviation below mean articulation rate, and more than 1.5 standard deviations below mean f0. A two-tailed comparison between the articulation rate of Birmingham 1 and that of the second slowest speaker, Plymouth 2, was significant, t(1092)=−17.58,pˇ.001 . Similarly, Birmingham 1 had a lower f0 than that of the second lowest f0 speaker, London 1, t(1117.8)=−49.39,p<.001 . These tests reinforce the impression that Birmingham 1 was a particularly slow and low-pitched speaker. For this reason, we reran our confirmatory and exploratory models with this speaker removed, to ensure that any observed effects were not heavily biased by this one prosodically outlying speaker. The descriptive statistics for the Birmingham accent excluding Birmingham Speaker 1 are shown in italics in the bottom row of Table 2.

When excluding Birmingham 1, the final confirmatory model contained the same set of fixed effects as when all speakers were included. That is, there were significant main effects of behaviour, $\chi^{2}(1)=269.2,p<.001$ ; game turn, $\chi^{2}(1)=10.61,p<.01$ ; and accent, $\chi^{2}(3)=18.47,p<.01$ , as well as significant interactions of Behaviour × Accent, $\chi^{2}(3)=19.85,p<.001$ and Behaviour × Game Turn, $\chi^{2}(1)=169.92,p<.001$ . In almost all cases, these effects reflect an identical pattern of results to the all-speakers model. The exception was the interaction of Behaviour × Accent: Post hoc tests showed the same significant pairwise comparisons as for the all-speakers model, but with an additional significant difference for the *mean* condition, where investments were significantly higher for London speakers compared with Plymouth speakers (p<.05) . However, we were again concerned about spurious differences arising from estimated marginal means. Specifically, the significant differences between SSBE and Birmingham speakers in the *generous* condition and between London and Plymouth speakers in the *mean* condition seemed likely to be misleading. We, therefore, ran two independent-samples *t* tests as detailed above to test these comparisons, and both were nonsignificant as expected: SSBE versus Birmingham for *generous*, t(66)=0.63,p=.53 ; London versus Plymouth for *mean*, t(80)=0.11,p=.91 . The Behaviour × Accent interaction without Birmingham 1 should, therefore, be interpreted as indicating an identical pattern of results to the all-speakers model.

For the exploratory analysis, the forward stepwise procedure resulted in a final model with only a main effect of articulation rate alongside the baseline effects from the final confirmatory model. This main effect indicated that a higher articulation rate was associated with lower investments, $\chi^{2}(1)=3.95,p<.05$ . Unlike the all-speakers exploratory model, there were no effects related to mean f0. This indicates that what appeared to be an f0 effect on investments with the full data set (higher f0 eliciting higher investment) was actually mediated by articulation rate: The outlying Birmingham speaker’s very low articulation rate was paired with a low f0, but once a more circumscribed set of prosodic values are used in the analyses, the direct influence of articulation rate—within a typical range—emerged (see discussion below). There were also no effects related to f0 range or H1 – H2.

### Questionnaire

The questionnaire provided a comparison between a traditional, explicit measure of trust and the novel, implicit trust measure in the investment game. There were explicit questionnaire ratings of trustworthiness across 14 speakers of 10 accents. A one-way analysis of variance (ANOVA) revealed significant differences in trust attributions between accents: F(3,659)=28.45,p<.001 . Post hoc comparisons, using the Tukey HSD test, between the four virtual game partner accents show that London, Plymouth, and SSBE accents were rated more trustworthy than Birmingham (all p<.001 ), and that SSBE was rated more trustworthy than London (p<.001) and Plymouth (p=.031) . This is a similar pattern to the accent-contingent investments in the *mean* condition of the investment game, where Birmingham speakers received lower investments than any other accent.

**Figure 4.**
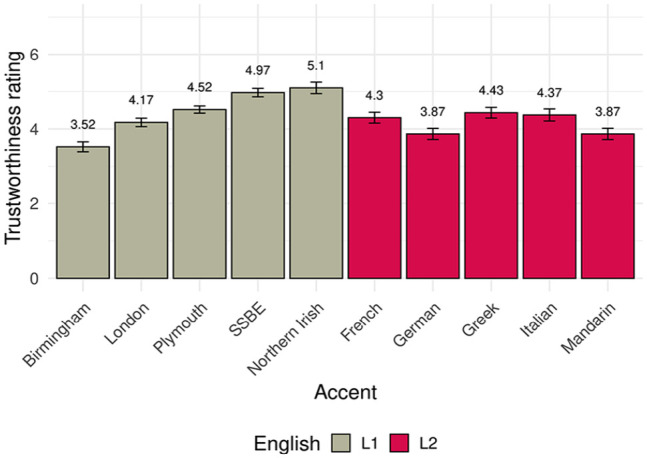
Mean trustworthiness rating of the 10 English accents played in the postexperiment questionnaire. Mean ratings are indicated above the bars, and error bars represent standard error.

It should be noted that participants did not rate the same speakers that they had heard in the game, but two other speakers with the same accent. These explicit trust ratings are in broad agreement with prior literature, with prestigious accents such as SSBE at the higher end of the trust scale, urban accents such as Birmingham (but not London—see below) at the lower end, and accents with rather more rural associations, such as Plymouth, in the middle (Bishop et al., 2005). Furthermore, in line with Lev-Ari and Keysar (2010), speakers of English as a second language overall received lower ratings than native English speakers: average trustworthiness rating of L1 speakers = 4.38; L2 speakers = 4.18; t(937.8)=2.32,p=.021 . As mean trustworthiness ratings shown in Figure 4 indicate, however, some native English accents received lower ratings than second language accents.

Despite listeners’ noted difficulties in localising accents (e.g., Goggin et al., 1991), participants were above chance in identifying the SSBE, Plymouth, and Birmingham accents (65.8%, 37.8%, and 41.5%, respectively), but not the London accent (1.2%). A post hoc examination of the influence of the participant’s own accent on investments with virtual partners with the same or different accent was not significant, $\chi^{2}(1,N=82)=0.04,p=.83$ , suggesting that any “accent loyalty” effect (Giles, 1970) was not a decisive influence on differential investment behaviour here, although more controlled studies would be required to distinguish the diverse potential effects of accent identity, familiarity, and other sociolinguistic factors on trustworthiness judgements.

Finally, we note that in questionnaire ratings of speakers with the same accent as an earlier game partner (i.e., the four trust game accents), the behaviour that raters experienced of that similarly accented partner did not have an effect on explicit trustworthiness, F(1,659)=1.68,p=.19 . That is, accents associated with *generous* game partners were not rated higher in the questionnaire, and accents associated with *mean* game partners were not rated lower.

## Discussion

Implicit trustworthiness attributions were modulated by the voice characteristics of a simulated game partner. As shown by higher investments, the standard accent—SSBE—was implicitly trusted more than regional accents overall, although the *generous* versus *mean* behaviour of the game partner interacted with the accent, as discussed below. The overall positive evaluation of standard accents is in line with previous questionnaire-based findings (Bishop et al., 2005; Giles, 1970). The current experiment has additionally shown that the accent effect is persistent over time and survives trust-relevant experience. At the start of the game, participants have no experience of the trustworthiness of their game partner, and differential judgements must rely upon preexisting stereotypes. It would be reasonable to expect that the effect of accent diminishes as the game progresses, as the stereotypes are overwritten by experience. This was not the case, however: Thus, we conclude that the influence of accent-related trust attribution is independent of experiential trust arising from reinforcement learning.

We also found that implicit trust attributions to different accents varied according to how the game partner was behaving. For example, participants made the greatest investments to the *generous* SSBE- and Birmingham-accented game partners, but the smallest ones to the *mean* Birmingham-accented partners. That is, while the Birmingham speakers received high investments in the *generous* condition, they received the lowest investments in the *mean* condition. One interpretation of these results is that when the speaker’s behaviour in the game becomes apparent, the subsequent investment patterns could reflect a reward or a penalty, according to whether the participant’s initial accent-based impression was congruent or incongruent (respectively) with observed behaviour. Such effects have been observed for faces. For example, Chang et al. (2010) reported that partners in an iterated investment game who initially appeared trustworthy and subsequently behaved trustworthily prompted the largest investments overall. Moreover, Wilson and Eckel (2006) report a “beauty penalty,” with lower returns for apparently trustworthy partners whose initial investments did not live up these expectations—see also Andreoni and Petrie (2008) and Solnick and Schweitzer (1999) for other examples of “beauty penalties” using different economic games. In this study, participants form an impression of a speaker’s trustworthiness upon hearing them for the first time—Round 1 in the investment game—but the actual trustworthiness of the speaker, in the form of the simulated *generous* and *mean* behaviours, refines this first impression. This modification appears contingent on whether the perceived and actual trustworthiness match or not. Thus, listeners might have formed a first impression of trustworthiness upon hearing the Birmingham speakers for the first time, subsequently reinforced in the *generous* condition but punished in the *mean* condition, when their behaviour was perceived to be incongruent. Such a “congruency effect” could apply to previous findings regarding SSBE and Liverpool speakers as well (Torre et al., 2015, 2018). Exactly when and how stereotype/behaviour congruency has an impact likely depends on other contextual and idiosyncratic factors yet to be fully determined: Unlike Torre et al. (2015), for example, we found no congruency effect for the SSBE speakers. And regardless of these nuances, accent exerts a persistent influence on trustworthiness judgements in the face of experience.

Regarding prosody, we initially found that higher f0 was associated with increased trustworthiness. This is consistent with Imhof’s (2010) finding that higher f0 was associated with higher agreeableness. The greater trustworthiness of voices with higher pitch may be related to “Size/Frequency Code” theory (Ohala, 1983), based on higher f0 being generally indicative of a smaller larynx and hence smaller body size. As a consequence, we tend to associate lower f0 with dominance and aggressiveness, and higher f0 with friendliness and cooperativeness (Hirschberg, 2002). By default, listeners might be expected to attribute trustworthiness to a speaker who is perceived as friendly rather than dominating, at least for the female speakers used in our study, although there is evidence that f0 effects interact with speaker gender (e.g., Montano et al., 2017).

However, this effect of f0 disappeared once one Birmingham-accented speaker with a substantially lower f0 and slower articulation rate was removed; instead, we observed an effect of articulation rate, with a lower rate being associated with higher investments, congruent with Niebuhr et al.’s (2016) proposal that slow rate might be a component of charismatic/persuasive speech. Given that the effect of rate emerges only when the outlyingly slow speaker is removed, this suggests that articulation rate has an inverted-U-shaped relationship with investment. In other words, a very low articulation rate led to lower apparent trustworthiness, which in this case gave rise to an apparent effect of f0 due to Birmingham 1’s particularly low f0. However, within a normal range of articulation rates (i.e., without Birmingham 1), a higher rate in fact led to lower trustworthiness. Moreover, the higher trustworthiness of voices with a slow articulation rate could be interpreted in terms of the “Effort Code,” which postulates that careful pronunciation of speech can signal cooperativeness (Gussenhoven, 2002). Thus, speaking at a slower rate might signal that the speaker is willing to sacrifice production efficiency and increase articulatory effort to aid listeners (see also hypo- vs. hyper-articulation theory, Lindblom, 1996). Manifestly, idiosyncratic speech characteristics, beyond perceived regional accent, also influence implicit judgements of trustworthiness.

Finally, it is worth noting that questionnaire ratings of speakers with the same accent as an earlier game partner were unaffected by the game partner’s behaviour. In other words, voices with a similar accent to a *generous* game partner were not rated more highly, nor were accents associated with *mean* game partners rated lower.

In a previous study (Torre et al., 2015), the behaviour assigned to the virtual player had an effect on the subsequent trustworthiness ratings of their accent, as exemplified by samples of that player’s voice. In this study, the exemplars provided for accent ratings were not spoken by the virtual players, but by speakers previously unknown to the participants. Here, we found that the earlier behaviour of the similarly accented virtual players had no effect on the trust ratings of the accents. This incidentally reinforces the finding that accent-related trust-judgements are remarkably persistent, even in the face of behavioural evidence about trustworthiness. Moreover, the implicit and dynamic nature of our experimental paradigm—the “repeated investment game”—allows us to assess how experience influences trusting behaviour over time, which traditional questionnaires do not afford, at the same time resulting in a more engaging experience for the study participants (Sailer et al., 2017).

## Conclusion

Voices provide cues which are used by listeners to make trustworthiness judgements. Accents have long been shown to influence first impressions of an individual’s trustworthiness, for reasons related to social stereotyping and with likely origin in the evolution of language as a social medium. Questionnaire methods have, however, only provided explicit and static measures of the influence of accent on trust attributions. The novel method used here provides implicit, repeated measures of trust, allowing us to examine how trust attributions change over time, as participants experience the actual trustworthiness of their game partners.

We showed that participants’ investments to a virtual partner, an implicit indicator of trust, were affected by various vocal features. In particular, slower speech rates were trusted more. Another strong effect was found for accent: Certain accents, in particular a non-regional standard associated with higher social class (SSBE), were found to influence investments. `Most critically, this accent influence was persistent over time, showing that voice-based stereotypes maintain their influence despite evidence of a speaker’s trustworthiness.

## Supplemental Material

sj-docx-1-qjp-10.1177_17470218231211549 – Supplemental material for The irrepressible influence of vocal stereotypes on trust Supplemental material, sj-docx-1-qjp-10.1177_17470218231211549 for The irrepressible influence of vocal stereotypes on trust by Ilaria Torre, Laurence White, Jeremy Goslin and Sarah Knight in Quarterly Journal of Experimental Psychology
