# Supplementary material for: The irrepressible influence of vocal stereotypes on trust
Source: Q J Exp Psychol (Hove). 2023 Nov 28;77(10):1957–66. doi: 10.1177/17470218231211549 (PMC11448076; doi:10.1177/17470218231211549)
Supplement: sj-docx-1-qjp-10.1177_17470218231211549 – Supplemental material for The irrepressible influence of vocal stereotypes on trust [file sj-docx-1-qjp-10.1177_17470218231211549.docx]

## Appendix A: Recorded utterances

### Block A

• Hello, nice to meet you. I am ready to play this game with you!

• Remember, there is potential for earning, if we both trust each other

• In my opinion, we should keep co-operating until the end

• Look at how fast the total money in the bank is growing!

• I’m going to return more money now, if you invest more as well

• There’s no gain in investing or returning nothing, we shouldn’t do that

• My strategy is clear: always return part of the investment

• I have been a bit mean with my returns, I will give you more from now on

• I will demonstrate that you can trust me, just as I trust you

• I think we can do better. Let’s try our best to get the bank growing

• The best strategy in this game is definitely to trust each other

• If we both invest in each other, the final reward will be bigger

• I think we can definitely go home with much more money than this

• We have to help each other out, it’s the only way to win the game

• I will return more money from this moment, this is a promise

• You have to trust me. I have every intention to repay your trust

• There is no point in keeping the money hidden under the mattress

• I trust you, and I will show you that you can trust me as well

• Let’s not undermine each other’s expectations, it would be a pity

• Sorry, I could have returned more money. But I can still do it now

### Block B

• Hello, nice to meet you. Let’s get started with the investment game!

• I have already thought of a strategy, I hope it will work

• If I keep all your investment, you will not invest anymore

• I will not keep all the money that you invest, this is a promise

• Even if I return part of your investment, I will make a profit

• What I keep is good enough for me, I am not that greedy

• As long as co-operation continues, I’m happy to earn a bit less

• I am going to share everything, you have to believe me

• You have to trust me, in the same way that I am trusting you

• If we could talk face-to-face at the moment, you’d know that I’m being honest

• Don’t forget that we will make more money if we both share what we get

• I want you to keep investing, and that is why I keep returning

• The more money you invest, the more money the two of us will earn

• This was a low payback; I am going return more from now on

• The goal of the game is to earn as much money as possible

• The only way to earn is by always co-operating and investing

• If we want to raise our earnings, we have to invest in one another

• I’m going to return more money than this, provided you keep investing

• I am going to co-operate until the very end of the game

• Come on, we can do better than this in the game, we have to keep trying

### Block C

• Hello, welcome to the game. Let’s see how much money we can make.

• There’s only one way to win the game, and that is by always sharing

• I think we should try our best to share the money we are given

• I promise that I won’t let you down, and I’ll always return your money

• My returns are always going to be high, if you invest at each round

• I trust you, and I promise that your trust will be well repaid

• We both want to win, but the only way to win is by cooperating

• If we want to earn more and more money, we have to trust each other

• The best strategy to win this game is simple, and it’s called trust

• Let’s keep cooperating, and we will both benefit from this

• We should trust each other, and we will be rewarded at the end

• We should have a clear strategy: always help each other out

• The only way to earn is for you to invest, and for me to return

• I will return more money now, because I want you to keep investing

• The more money you invest, the more your bank and mine will grow

• You have to trust me, and rest assured that I will return your trust

• I am expecting you to share, because that’s exactly what I am doing

• If I can persuade you to invest more by returning more, I will do it

• There doesn’t have to be only one winner; we can both win the game

• I’m not going to keep your money, this won’t help me in the long run

### Block D

• Welcome to the investment game. I hope we will enjoy playing it.

• In my opinion, we should always invest in one another

• I think we can do better. I promise that I will not let you down

• If we both invest in each other, we will surely raise our earnings

• We could finish the game better off than this, if only we tried harder

• You have to trust that I’m going to cooperate until the last round

• We can both win the game, but we have to keep sharing our money

• I trust you, and I am sure that we can both benefit from each other

• I am not a greedy person, and I believe we should share these earnings

• We can earn more money than this, if we co-operate until the end

• I will return more of your investments, you have to trust me in this

• When the game ends, I promise that we will both be satisfied with the outcome

• Remember this: it’s not convenient for me to keep all your investments

• No matter the number of rounds, we should trust each other until the end

• There is no better tactic than to keep investing and returning

• I will show you that co-operation is the best option for us

• Let’s keep sharing, and our earnings will grow much bigger than they are now

• I promise that I am going to return more money from now on

• I will always return, because there’s no point in me doing otherwise

• If we want to see our funds growing, we have to share until the end
